# Mandatory Third-Party Exclusion of Individuals with Gambling Problems in Germany: Data from the OASIS Player Exclusion System

**DOI:** 10.1007/s10899-025-10427-6

**Published:** 2025-10-13

**Authors:** Gerhard Meyer

**Affiliations:** University of Bremen, Grazer Strasse 2, Bremen, 28359 Germany

**Keywords:** Gambling, Gambling disorder, Self-exclusion, Third-party exclusion, Public health, Legal regulations

## Abstract

While self-exclusion for individuals who gamble is widely recognized and implemented as an important consumer protection measure worldwide, third-party exclusion, initiated by relatives or gambling providers, is currently only available in a few countries. The aim of this article is to provide a timely descriptive evaluation of the exclusion registry in Germany, where third-party exclusion is mandatory by law, in relation to different types of gambling and the frequency of use by gambling providers. The relevant authority has provided data from licensed providers, broken down by self-exclusion and third-party exclusion. The majority of exclusion requests in 2024 (*N* = 303,876) were made through self-exclusion. Specifically, 96.8% of all exclusions were initiated by the players themselves, while only 3.2% resulted from third-party requests. The lowest proportions of third-party exclusions were observed in the sectors of gambling halls with German-style slot machines (0.7%), virtual slot machines (1.1%), and online poker (1.5%). The low proportion of third-party exclusions may be due to a conflict of interest, as gambling providers often generate significant revenue from individuals with gambling problems, making this group a lucrative target market. However, preliminary empirical evidence suggests that third-party exclusions have positive effects, showing comparable rates of abstinence and reduced gambling behavior to those who self-exclude. Mandatory third-party exclusions help minimize harm and represent a valuable addition to public health strategies. Nevertheless, further research is needed to expand the limited database, and the low use of third-party exclusions by providers calls for stronger regulatory oversight.

## Introduction

Globally, gambling policies have primarily focused on interventions aimed at individuals who gamble, rather than addressing harmful industry practices, products, and systems (Ukhava et al., 2024). Among the most common individual-focused measures is self-exclusion. Gambling operators are required to offer self-exclusion programs as a means for individuals with gambling problems to avoid further gambling. These programs have proven to be an effective component of public health strategies aimed at minimizing gambling-related harm (Devault-Tousignant et al., 2023; Drawson et al., 2017; Gainsbury, 2014; Kotter et al., 2019; McMahon et al., 2019; Motka et al., 2018). Recently, the Lancet Public Health Commission on Gambling also recommended self-exclusion as an essential consumer protection measure (Wardle et al., 2024). However, research indicates that self-exclusion programs are underutilized by people with gambling disorder and are not always fully effective in preventing individuals from gambling at venues where they have excluded themselves (Gainsbury, 2014; Kraus et al., 2022). Structural barriers, such as the need to appear in person to self-exclude, limited coverage (e.g., restricted to specific venue types like casinos and online platforms), and exclusions that do not include foreign operators, often make these programs easy to circumvent (Kraus et al., 2024).

A few countries have developed more comprehensive systems of measures targeting gambling, which extend beyond individual exclusions. In addition to self-exclusion, third-party exclusion by family members is available in Belgium^1^, Singapore^2^ (Goh et al., 2016), and New Zealand^3^. Alongside provider-initiated exclusions, third-party exclusions are required by law in Switzerland, Austria, and Germany.

In Switzerland, the Federal Gambling Act mandates that gambling operators exclude individuals from gambling if there is evidence that they are accumulating excessive debt, placing disproportionate bets relative to their financial means, or experiencing other disruptions due to their gambling behavior (Lischer et al., 2023). This exclusion program covers multiple venues, meaning that banned individuals are excluded from land-based casinos, licensed online casinos, online lotteries, and sports betting. In 2021, 12,133 new exclusions were issued, bringing the total number of exclusions to 79,917 by the end of that year. However, data differentiating between self-exclusion, third-party exclusion, and types of gambling is not available.

In Austria, operators of games of chance at the federal level (e.g., casinos, video lottery terminals, and online platforms) are required by the Austrian Gambling Act (GSpG) to report data on third-party and self-exclusion, loss and visit limits to the Federal Ministry of Finance. According to Section 25 of the Gaming Act, casinos must refuse entry to individuals whose gambling behavior puts their livelihood level at risk. This exclusion is the final step after various graduated measures, and independent credit checks are required to clarify the situation. Operators at the state level (such as those for slot machines) are subject to similar reporting obligations, although these are governed by state law. Currently, there is no central exclusion registry that enables a comprehensive view of bans across all providers and gambling forms (Puhm et al., 2018).

In contrast, Germany has a centralized exclusion system, the OASIS (“Online Abfrage Spielerstatus”) player exclusion system. The establishment of this central exclusion file was outlined in the State Treaty on Gambling (GlüStV) of 2012 and updated with the current version of the GlüStV as of July 1, 2021. Prior to this, exclusion options were already available in casinos, where a self- and third-party exclusion system had been in place since 1981 under a private law agreement. The 2008 version of the GlüStV extended these requirements to state lottery companies (DLTB, German Lotto and Toto Block), obliging them to implement exclusion systems for high-risk sports betting and lottery games (e.g., Oddset, Toto, Keno). Under the 2021 GlüStV, almost all licensed providers of land-based and online gambling are now required to connect to the exclusion registry, with a few exceptions (e.g., lotteries with no more than two draws per week, prize savings, and specific types of horse betting).

Providers of games of chance are required to exclude individuals who request self-exclusion or those identified by staff or reported by third parties. This applies when there is reason to believe that a person may be at risk of gambling addiction, accumulating excessive debt, failing to meet financial obligations, or placing stakes that are disproportionate to their income or assets (third-party exclusion, § 8a, 1, GlüStV 2021). Staff must be trained in the early detection of individuals with gambling problems. Indications of excessive debt can, e.g., be obtained through inquiries with the General Credit Protection Agency (SCHUFA). The person to be excluded has the opportunity to submit a statement after the third-party exclusion request has been received. Providers must assess the risk of addiction both during registration and before each gambling session. Players are only allowed to participate if the system returns a confirmation that the individual is not banned. Excluded individuals are also prohibited from receiving gambling-related advertisements. The exclusion period lasts for a minimum of one year, unless the person requesting self-exclusion applies for a shorter period, which cannot be less than three months. A request to lift an exclusion must be submitted in writing to the competent authority (Darmstadt Regional Council). This procedure marks a significant change, as previously, the gambling provider who imposed the ban was solely responsible for deciding whether to lift it. Under the current system, authorities are now required to inform both the gambling providers and the relatives who initiated third-party exclusions about the application to lift the ban and the possibility of submitting a new exclusion request (§ 8b, 4).

There is also an option of a 24-hour exclusion that can be triggered by the “panic button” in sports betting, online casino games, online poker, and virtual slot machines on the internet (§ 6i, 3). Providers of these gambling formats must, at their own expense, implement an automated system based on scientific findings and algorithms to detect early signs of gambling addiction (§ 6i, 1).

Not only in Germany has the exclusion system been further developed in recent years. Internationally, self-exclusion is now fully digital, with real-time ID checks, national or even cross-border registration, flexible time periods, and integration with treatment services. However, not all at-risk individuals recognize their own problem or act accordingly (lack of insight, limited self-control, Meyer & Bachmann, 2017). The third-party exclusion aims to close protection gaps that exist when only voluntary self-exclusion options are available. It complements self-exclusion in cases of relapse risk, provides opportunities for early intervention, allows relatives to exclude players, and places greater responsibility on gambling providers. In this context, Germany’s central exclusion system covering all types of gambling may serve as a model for other countries.

The aim of this study is to provide a timely description of data of the German OASIS exclusion system, by year, self- vs. third-party exclusions, and gambling type, submitted to the competent authority. Specifically, the article aims to assess the frequency of third-party exclusions by gambling providers in relation to different gambling formats. The available data offer valuable insights that can be used worldwide to optimize harm reduction in the field of gambling.

### Data from the OASIS Player Exclusion System

The Darmstadt Regional Council, as the responsible authority, provided the OASIS data for this article. The OASIS exclusion file stores relevant data on self-exclusion and third-party exclusion to enable the management and enforcement of such bans.

The following data is stored:

- date and place of birth of the player,
- username, date of account creation, or other relevant data for identifying the player,
- address,
- duration of the exclusion,
- reason for the player’s exclusion,
- provider or reporting authority.

The information in the OASIS exclusion file is not accessible to third parties or other information systems such as General Credit Protection Agency (SCHUFA), credit institutions, or banks.

The exclusion file provided by the administration for this article includes data on licensed providers of games of chance, such as: (1) operators of casinos, (2) organizers of online poker, (3) providers of virtual slot machines on the internet, (4) organizers of lotteries held more than twice a week, (5) sports betting organizers, (6) sports betting brokers, (7) online horse racing betting operators, (8) gambling arcade operators (with German-style slot machines^4^), (9) operators of German-style slot machines in pubs, and (10) the competent government agency. The file contains information on self-exclusions initiated by players and third-party exclusions by relatives or providers, along with the duration of each exclusion. However, the file does not provide details on the distribution of third-party exclusions by relatives or providers, nor does it include personal information or reasons for the exclusion.

### Statistical Analysis

The ratios of self- and third-party exclusions across different types of gambling were analyzed for significant differences using odds ratios and 95% confidence intervals (Dean et al., 2013).

## Results

Table 1 presents the number of exclusion requests received between 2020 and 2024, broken down by individual gambling providers (including the responsible authority) and categorized by self- and third-party exclusions. Self-exclusions are temporary or indefinite, while third-party exclusions are always indefinite.

**Table 1:** Self- and third-party exclusion for different providers of gambling in the OASIS player exclusion system. (Data for 2020, the year before the introduction of OASIS, from the German Exclusion Database)

**Gambling Provider and Government Agency** ^**a**^	**2020** ***N*** ** (%)**	**2021** ^**b**^ ***N*** ** (%)**	**2022** ***N*** ** (%)**	**2023** ***N*** ** (%)**	**2024** ***N*** ** (%)**
Provider of Casinos (Table Games/Slot Machines)					
Self-Exclusion	35,796 (84.7%)	39,734 (85.9%)	38,782 (86.6%)	42,330 (88.0%)	45,530 (88.9%)
Third-Party Exclusion	6,447 (15.3%)	6,522 (14.1%)	6,023 (13.4%)	5,798 (12.0%)	5,666 (11.1%)
**Total**	**42**,**243 (100%)**	**46**,**256 (100%)**	**44**,**805 (100%)**	**48**,**128 (100%)**	**51**,**196 (100%)**
Organizer of Online-Poker					
Self-Exclusion	-	-	-	2,605 (98.4%)	4,287 (98.5%)
Third-Party Exclusion	-	-	-	43 (1.6%)	67 (1.5%)
**Total**	-	-	-	**2**,**648 (100%)**	**4**,**354 (100%)**
Organizer of Virtual Slot Machines					
Self-Exclusion	-	-	3,380 (98.%)	25,231 (98,8%)	39,795 (98.9%)
Third-Party Exclusion	-	-	39 (1.1%)	314 (1.2%)	461 (1.1%)
**Total**	-	-	**3**,**419 (100%)**	**25**,**545 (100%)**	**40**,**256 (100%)**
Provider of Lotteries (more than twice a week)					
Self-Exclusion	2,015 (87.6%)	2,113 (86.9%)	2,002 (85.6%)	1,931 (84.9%)	1,889 (82.8%)
Third-Party Exclusion	285 (12.4%)	319 (13.1%)	337 (14.4%)	344 (15.1%)	393 (17.2%)
**Total**	**2**,**300 (100%)**	**2**,**432 (100%)**	**2**,**339 (100%)**	**2**,**275 (100%)**	**2**,**282 (100%)**
Organizer of Sports Betting					
Self-Exclusion	2,059 (87.1%)	29,853 (96.1%)	46.033 (96.6%)	58.442 (97.3%)	70,795 (97.6%)
Third-Party Exclusion	306 (12.9%)	1,223 (3.9%)	1,636 (3.4%)	1,602 (2.7%)	1,717 (2.4%)
**Total**	**2**,**365 (100%)**	**31**,**076 (100%)**	**47**,**669 (100%)**	**60**,**044 (100%)**	**72**,**512 (100%)**
Broker of Sports Betting					
Self-Exclusion	-	198 (95.2%)	408 (94.2%)	883 (95.6%)	1,368 (96.3%)
Third-Party Exclusion	-	10 (4.8%)	23 (5.3%)	41 (4.4%)	53 (3.7%)
**Total**	-	**208 (100%)**	**431 (100%)**	**924 (100%)**	**1**,**421 (100%)**
Organizer of Horse Betting (Online/Bookmaker)					
Self-Exclusion	40 (100%)	56 (100%)	72/6 (100/86%)	104/9 (100/90%)	100/16(100/88.9%)
Third-Party Exclusion	-	**-**	-/1 (-/14%)	-/1(-/10%)	-/2(-/11.1%)
**Total**	**40 (100%)**	**56 (100%)**	**72/7 (100/86%)**	**104/10 (100%)**	**100/18 (100%)**
Operator of Gambling Halls (German-Style Slot Machines)					
Self-Exclusion	-	23,441 (98.6%)	45,813 (99.0%)	75,080 (99.3%)	89,686 (99.3%)
Third-Party Exclusion	-	342 (1,4%)	452 (1,0%)	563 (0,7%)	630 (0.7%)
**Total**	-	**23**,**783 (100%)**	**46**,**265 (100%)**	**75**,**643 (100%)**	**90**,**316 (100%)**
Operator of German-Style Slot Machines in Pubs					
Self-Exclusion	-	114 (100%)	460 (98,3%)	969 (98,4%)	1,372 (98.1%)
Third-Party Exclusion	-	-	8 (1,7%)	16 (1,6%)	26 (1.9%).
**Total**	-	**114 (100%)**	**468 (100%)**	**985 (100%)**	**1.398 (100%)**
Government Agency					
Self-Exclusion	-	2,924 (99,3%)	15,057 (98,5%)	28,166 (97,7%)	39,197 (98.0%)
Third-Party Exclusion	-	20 (0,7%)	227 (1,5%)	658 (2,3%)	814 (2.0%)
**Total**	-	**2**,**944 (100%)**	**15**,**284 (100%)**	**28**,**824 (100%)**	**40**,**011 (100%)**
Total					
Self-Exclusion	39,910 (80,7%)	98,433 (92,1%)	152,013 (94,6%)	235,750 (96,2%)	294,044 (96.8%)
Third-Party Exclusion	7,038 (19,3%)	8,436 (7,9%)	8,746 (5,4%)	9,380 (3,8%)	9,832 (3.2%)
**Total**	**46**,**948 (100%)**	**106**,**869 (100%)**	**160**,**759 (100%)**	**245**,**130 (100%)**	**303**,**876 (100%)**

Source: Darmstadt Regional Council (OASIS, Dez. II 24.1)

^a^Data can’t be assigned to specific forms of gambling

^b^Data for 2021 since July 1, 2021

With the introduction of the OASIS exclusion file in July 2021, the number of ban requests (including lifted exclusions) increased by 547.3%, rising from 46,948 in 2020 to 303,876 in 2024, compared to the previous year, by 24%. The highest number of exclusions (90,316, or 29.7%) were recorded for gambling arcades with German-style slot machines, followed by sports betting organizers (23.9%) and casino operators (16.8%).

The majority of exclusion requests were for self-exclusion, with 96.8% of the total (*N* = 294.044) initiated by players themselves, while only 3.2% (*N* = 9,832) were third-party exclusions. The proportion of third-party exclusions was significantly lower in gambling halls with German-style slot machines (0.7%) compared to casinos (11.1%) and lotteries with more than two weekly draws (17.2%). The difference in probability between gambling halls and casinos was a factor of 16 (OR = 15.87; 95% CI = 14.60–17,24), and between gambling halls and lotteries, it was a factor of 25 (OR = 24.69; 95% CI = 21.62–28.19). Additionally, the lowest proportions of third-party exclusions were found in virtual slot machines (1.1%) and online poker (1.5%).

In most cases (reference date: May 2023) exclusions were indefinite (60.4%), followed by bans lasting 1 to 2 years (13.0%), and those lasting 3 to 6 months (8.5%) (Table 2).

**Table 2:** Duration of exclusion. (Data refer to the reference date of May 16, 2023)

**Duration**	***N***
More than 10 years	12,148
5 to 10 years	4,518
1 to 2 years	36,213
6 to 12 months	14,398
2 to 5 years	19,175
3 to 6 months	23,501
Unlimited	167,785
**Total**	**277,738**

Further data from the authority regarding player exclusions show that the short-term “panic button” feature (a 24-hour ban) was activated 546,213 times by gambling participants in 2024. The number of exclusions lifted during this period was 41,657. Providers connected to the OASIS system submitted approximately 1.124 billion requests to the authority to check the ban status of individuals.

## Discussion

The data from OASIS reveal a significant 547.3% increase in the number of exclusion requests following its introduction. This surge can likely be attributed to the inclusion of nearly all legal land-based and online games of chance that carry a higher risk potential. Additionally, the easier process of lifting exclusions upon the player’s request has been cited as a contributing factor (Quack, 2024).

The highest rates of exclusions are seen in gambling formats such as German-style slot machines, sports betting, and casino games (including both table games and slot machines). These forms of gambling have relatively low participation rates in recent prevalence studies (Buth et al., 2024), with participation at 1.9%, 1.1%, and 1.0%/0.5%, respectively. For example, 75,050 players at German-style slot machines had self-excluded in 2023, which corresponds to 6.8% of those who played on the slot-machines in that year (1.1 million). At the same time, these formats also have a high proportion of players with gambling disorder (25.5%, 31.8%, and 21.4%/21.8%, respectively). By comparison, the participation rate for the lottery ‘6 out of 49’ is 19.8%, with 5.9% of participants affected by a gambling disorder. The exclusion rates further indicate the high risk potential of gambling activities with high event frequency (cf. Allami et al., 2021).

In this context, it is worth noting that the acceptance of an exclusion system among the German population is high, as shown by data from the current prevalence study (Buth et al., 2024). A total of 75.9% of respondents support this measure for risky forms of gambling, rising to 79.9% among those who have gambled in the past 12 months.

In relation to the general adult population, 0.36% of the German population had been excluded from gambling by the end of 2024. A systematic review of self-exclusion at the international level by Biker et al. (2023) found a median prevalence of 0.26% (95% CI 0.16–0.43), placing the German figure at the upper end of this range.

At 96.8%, most of the OASIS exclusion requests are self-exclusions, with the majority being indefinite (60.4%). The 3.2% of third-party exclusions, however, are not broken down by the sources of the request—whether by relatives or gambling providers. Data from 2008 to 2010 show that almost half of the bans in casinos and for high-risk lotteries were initiated by both groups (Meyer, 2012). The question remains why so few third-party exclusions are requested, despite gambling providers being legally required to exclude players if relevant information is available (e.g., staff observations or machine learning algorithms). According to a representative study from 2023, 2.4% of respondents aged 18 to 70 show signs of gambling disorder (around 1.38 million people, Buth et al., 2024), with 6.1% exhibiting risky gambling behavior. Although not all players excluded via self- or third-party exclusion are individuals with gambling problems or a gambling disorder, however, the proportion among self-excluded players is high, ranging from 61 to 95% worldwide (Gainsbury, 2014; Kotter et al., 2019). A comparison of self- and third-party excluded individuals shows no significant differences in their characteristics (Kotter et al., 2018). Furthermore, self-excluded players cite annoyance with operators as a reason for exclusion (Hayer & Meyer, 2011).

The available descriptive data from the OASIS exclusion system do not provide an explanation for why providers often fail to implement third-party exclusions. One possible explanation is a conflict of interest. A significant portion of gambling revenue comes from individuals with gambling problems, making them a profitable target group (Fiedler et al., 2019). In fact, research on German casinos found that individuals with gambling problems or a gambling disorder account for around 60% of the turnover (Fiedler, 2015). Despite strict German laws requiring casino access controls and identification checks, 26% of excluded individuals were still able to continue gambling (Kotter et al., 2018). Mystery shopping research in gambling halls shows that only 7% of players exhibiting suspicious gambling behavior were approached by employees (Hayer et al., 2020). In 16% of access attempts by test players, no entry checks were carried out, and in 28% of gambling halls, the players were allowed to gamble despite being excluded. Loy et al. (2020) also highlight potential conflicts faced by employees, who may be reluctant to intervene with regular customers due to personal relationships or fear of conflict, especially since the most profitable players often ensure continued employment for the staff. This conflict of interest can hinder proactive action, along with unclear responsibilities and a lack of the necessary skills (Hayer et al., 2020). As a result, staff tend to engage with individuals with gambling problems only in extreme cases, such as with aggressive or disruptive customers (Hing et al., 2013).

There is also a notable difference between state and private gambling providers. The proportion of third-party exclusions is higher in casinos and lotteries than in gambling halls, possibly because almost half of the casinos (30 of 65) and lotteries are state-operated, which comes with a stronger obligation to follow the rules. By contrast, gambling halls are privately owned. However, even in casinos, third-party exclusions remain low. In 2010, bans based solely on staff observations covered just 0.4% of all acute individuals with a gambling disorder (Fiedler, 2015).

The conflict of interest arising from the financial benefits derived from people with gambling disorder underscores the need for stronger state oversight, along with sanctions such as license revocation (Loy et al., 2020). However, this applies only to licensed services. Given that illegal gambling, particularly online, is easily accessible to excluded players, more consistent enforcement is required in this area, which remains insufficient (Kraus et al., 2022). This includes measures like payment or internet access blocking. A lack of regulatory control is also evident with legal offers, especially in contexts where governments have vested interests in gambling revenues (Wardle et al., 2024). The effectiveness of regulation is further undermined by the limited resources available to regulatory bodies, making it difficult for them to keep up with technological advancements in the gambling industry.

Initial empirical results from Kotter et al. (2018) show that players excluded by third parties (via imposed bans) exhibit similar abstinence and reduced gambling behaviors as those who self-excluded voluntarily. This finding challenges the assumption that voluntary self-exclusion is the only route to behavioral change. More research is needed to understand why those who are third-party excluded did not choose to self-exclude in the first place. It is also important to explore which factors influence the effectiveness of imposed exclusions, whether from operators or relatives, as well as the severity of the gambling problems and the individual’s self-awareness (Kotter et al., 2018).

In Swiss casinos, self-exclusions are lifted more frequently than imposed exclusions (Lischer & Schwarz, 2018). However, no empirical data are available on the differences in effectiveness between family-imposed and operator-imposed exclusions. In Singapore, family members report positive outcomes from family-imposed bans (Goh et al., 2016).

When gambling providers fail to comply with their legal obligations to exclude visibly individuals with gambling problems, those affected are in a strong legal position to pursue damages. Under the GlüStV 2008, the highest court in Germany ruled that a casino must compensate a player for losses incurred after it failed to thoroughly review an application to lift a self-exclusion (Federal Court of Justice, 20.10.2011 - III ZR 251/10). A ban can only be lifted if there is sufficient evidence that the player is no longer at risk of gambling addiction and can participate in gambling in a controlled manner. While the current legal situation no longer mandates checking the status of a lifted ban, providers are still responsible for detecting gambling addiction and identifying players at risk, as outlined in the GlüStV 2021.

In summary, mandatory third-party exclusion implemented by gambling providers represents a targeted harm reduction measure. It obliges providers to exclude individuals who are visibly at risk or already affected by gambling addiction. However, due to the providers’ inherent conflict of interest, the implementation rate remains low. Initial empirical findings suggest that its effectiveness in reducing gambling-related harm is comparable to that of voluntary self-exclusion. To validate and expand on these findings, further accompanying research is necessary, along with increased governmental oversight.

With the ongoing digitalization of the gambling industry, online gambling providers now have access to advanced technologies that allow for the early detection of at-risk behavior. These operators routinely collect extensive customer data via individual user accounts, enabling behavioral tracking over time. This data-driven monitoring presents opportunities to identify problematic behavior and to intervene accordingly. For example, Finkenwirth et al. (2021) analyzed data from self-excluded players in British Columbia, Canada, and used machine learning models to determine risk scores. The most predictive variable for self-exclusion was the variance in money bet per session. Similarly, a study by Hopfgartner et al. (2023), using player tracking data from three online gambling platforms across six countries, found higher odds for self-exclusion among users exhibiting certain behavioral patterns:

1. a high number of previous voluntary limit changes and self-exclusions,
2. frequent use of different payment methods for deposits,
3. a higher average number of deposits per session, and
4. engagement with a wider range of game types.

Despite these technological capabilities, digital infrastructures have so far been used primarily for personalized marketing and increasing user engagement (Wardle et al., 2024), rather than for effective harm reduction.

Given the rapid growth of online gambling, there is a pressing need for effective regulatory controls and coordinated international frameworks. Recommendations to improve third-party exclusion often overlap with those concerning self-exclusion (Kotter et al., 2019; Kraus et al., 2024; Lischer et al., 2023). For instance, gambling bans should be more actively promoted and linked to therapeutic services for those affected, such as counseling and treatment. A ban, while not an intervention in itself, can serve as a motivational catalyst. The development and strengthening of abstinence motivation - and the implementation of appropriate strategies - should be supported through accompanying counseling and therapy.

In this context, an online self-management program has been introduced as an alternative to in-person support for voluntarily self-excluded individuals (Yakovenko & Hodgins, 2021). This digital approach is easier to implement, reaches a broader population, and is grounded in evidence-based psychotherapeutic principles for treating gambling disorder. Financial counseling, particularly on debt-related issues, and community-based aftercare programs are also considered essential support services. For family members and other affected parties, improved guidelines and counseling services should be made more widely available and actively promoted.

In addition to early detection, training programs for gambling provider staff should increasingly focus on identifying and addressing at-risk individuals before, during (in special cases), and after gambling activities. These trainings must also transparently address the potential conflict of interest involved.

Third-party exclusion should be integrated as a core consumer protection measure within broader public health strategies. Nevertheless, it represents just one component of a comprehensive harm reduction approach. According to the Lancet Public Health Commission on Gambling (Wardle et al., 2024), a robust and forward-thinking prevention strategy should, in addition to the already included self-exclusion, be complemented by the following measures:

1. Placing public health and well-being above competing economic interests in policy decisions,
2. Reducing population-level exposure and access to gambling through restrictions on availability, promotion, advertising, and sponsorship,
3. Providing affordable, universal support and treatment services for gambling-related harm,
4. Protecting children and young people through minimum age requirements and mandatory identification,
5. Regulating gambling products based on their potential for harm, and.
6. Enforcing limits on gambling activity, including deposit and betting limits.

## Limitations

The descriptive data do not allow for conclusive inferences regarding causality or the external validity of the findings. A more comprehensive understanding of the role of third-party exclusion could be achieved through comparative analyses of self- and third-party-excluded gamblers with respect to their use of exclusion measures, the collection of longitudinal data from individuals with gambling problems, and the systematic inclusion of data provided by affected family members as well as employees of gambling operators.

The presented data on third-party exclusions in the OASIS exclusion system does not differentiate between exclusions initiated by relatives and those initiated by gambling providers. According to the responsible authority, no differentiated recording of submitted third-party exclusions is carried out.

## Conclusion

Available findings from clinical studies (Kotter et al., 2018) suggest that mandatory third-party exclusion by relatives and gambling providers is an effective harm-reduction measure, making it a valuable component of a public health strategy. However, as this study shows, there is a low use of third-party exclusions by providers in Germany which appears to be largely driven by conflicts of interest. To address this issue, stronger state oversight is necessary. Future research should further explore the positive effects of third-party exclusions and identify the factors contributing to their success. Additional recommendations for improvement include a greater emphasis on promoting exclusions, linking them to counseling and treatment options for affected players and their families, enhancing the qualifications of provider staff, and increasing the use of technologies such as machine learning for the early detection of at-risk gambling behavior, particularly in the online sector.

## Data Availability

The data used in this study was provided by the competent authority who authorize its release by the author. The Darmstatd Regional Council is currently working on making the file publicly accessible.

## Footnotes

1
https://www.gamingcommission.be/en/protection-of-players (accessed December 15th 2024).

2 In Singapore the state also automatically bars undischarged bankrupts and Singaporeans who are receiving various forms of financial assistance from the government.

3
https://www.dia.govt.nz/diawebsite.nsf/wpg_URL/Services-Casino-and-Non-Casino-Gaming-Exclusion-Order-(Problem-Gamblers)-Guidelines (accessed December 15th 2024).

4 German-style slot machines, which are permitted in amusement arcades and pubs, are subject to legal regulations intended to distinguish them from casino slot machines and to reduce their potential for harm and support addiction prevention. According to the Gaming Ordinance, stakes and winnings per game are limited to 20 cents and 2 euros respectively, losses and winnings per hour are capped at 60 euros and 400 euros respectively, and the minimum game duration is set at 5 s. However, through devious circumvention of these legal requirements via so-called point-based games, stakes of 4 euros per game, winnings of over 4,000 euros, and games every second become possible (Meyer, 2019). The trick behind the point-based game: It is not the actual game event—such as the spinning of the reels or the display of symbols—that is declared as the gaming unit, but rather a preceding or subsequent process of converting monetary amounts into equally valued points and back into money. This form of slot machine gambling generates the highest gross gaming revenue in the German gambling sector. amounting to 4.9 billion euros in 2024, almost four times as much as that of casinos (with regular forms of gambling). The assessment of the health and social risks associated with German-style slot machines, as well as potential changes to legislation, are currently the subject of political debate.
